# Supplementary material for: Acute buprenorphine exposure depresses neonatal respiratory chemoreflexes in the presence or absence of naloxone
Source: bioRxiv. 2026 May 17:2026.05.13.724975. Preprint. [Version 1] doi: 10.64898/2026.05.13.724975 (PMC13192603; doi:10.64898/2026.05.13.724975)
Supplement: Supplement 1 [file media-1.pdf]

Supplemental Material for:

Acute buprenorphine exposure depresses neonatal respiratory chemoreflexes in the presence or absence of naloxone

*Michael Frazure[1], Khushi Praveen[4], Elias Sitzmann[1], Emily Flanigan[1], and Ralph Fregosi[1,2,3]

Department of Physiology[1], Neuroscience[2], Anesthesiology[3], and Biomedical Engineering[4], University of Arizona, Tucson, AZ, 85724-5051, USA

*Corresponding Author: Michael Frazure, PhD
mfrazure@arizona.edu
1501 N Campbell Ave
College of Medicine 4224
Tucson, AZ, 85724-5051

Supplemental Figures: 3
Supplemental Tables: 1

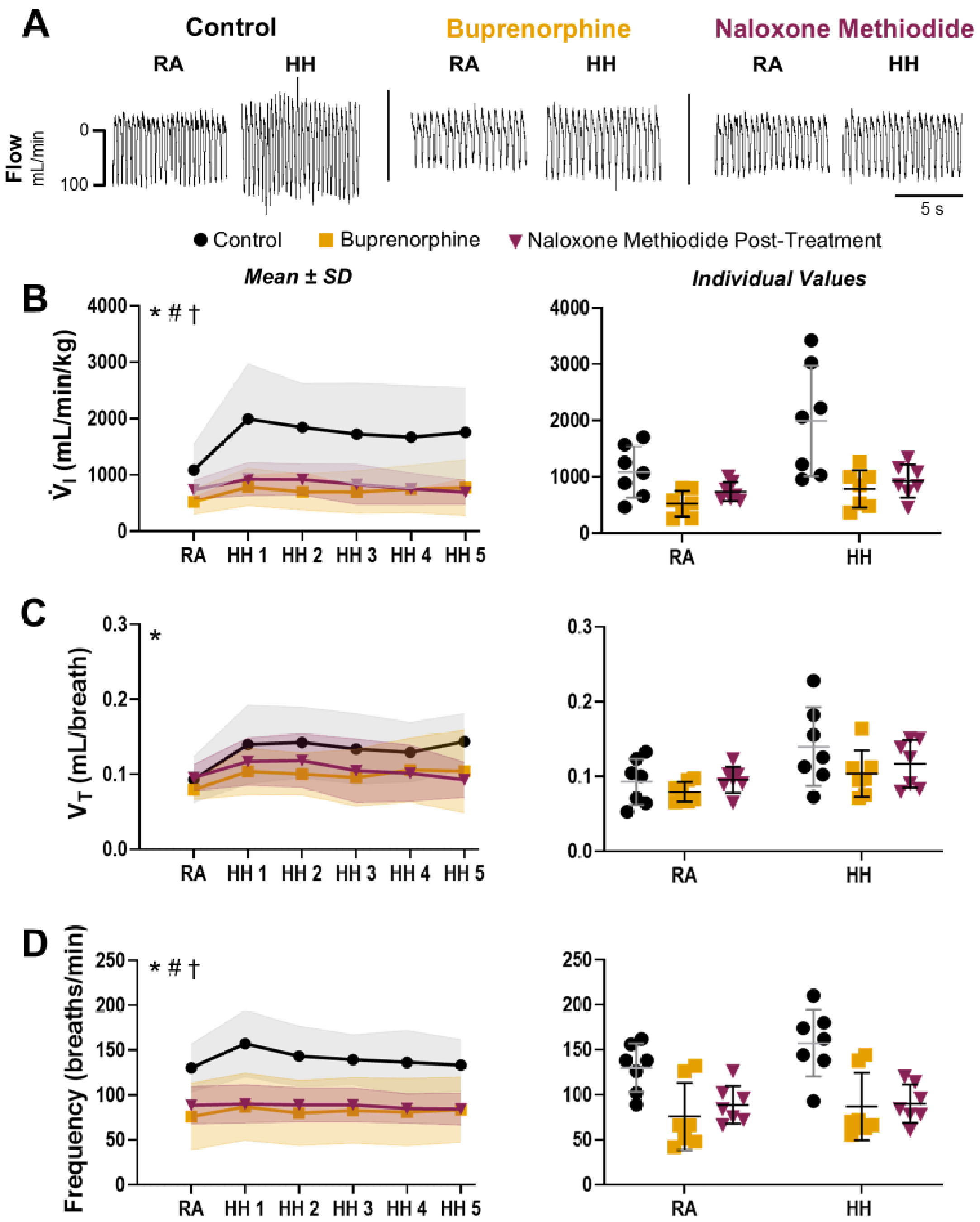

Supplemental Figure 1. Naloxone methiodide post-treatment does not reverse buprenorphine-induced respiratory depression. $\dot{V}_I$, V_T and breathing frequency were measured using dual-chamber plethysmography in neonatal Sprague Dawley rats (*n* = 7, 4 male; P4-5) during RA breathing and a 5-min HH challenge (HH1-5). Measurements were obtained before and after administration of buprenorphine, followed by peripherally restricted

naloxone methiodide. **Panel A:** Representative plethysmography traces illustrate the effects of gas mixture (RA, HH) and drug condition (Control, Buprenorphine, Naloxone Methiodide) on breathing. **Panels B–D:** Group data (left) and individual replicates (right) show the effects of drug and gas mixture on $\dot{V}_I$ (B), V_T (C), and breathing frequency (D). Two-way repeated measures ANOVA revealed significant effects of gas ($F_{1,6} = 20.18$, $p = 0.00414$), drug ($F_{2,12} = 12.99$, $p = 0.00675$), and a gas × drug interaction ($F_{2,12} = 13.30$, $p = 0.00432$) for $\dot{V}_I$. A significant effect of gas mixture ($F_{1,6} = 12.98$, $p = 0.0113$) was observed for V_T. Significant effects of gas ($F_{1,6} = 22.11$, $p = 0.00332$), drug ($F_{2,12} = 31.33$, $p < 0.0001$), and gas × drug interaction ($F_{2,12} = 7.89$, $p = 0.00922$) were observed for breathing frequency. Buprenorphine reduced resting ventilation and markedly attenuated the ventilatory response to HH via blunted V_T and elimination of the frequency response. Naloxone methiodide failed to restore $\dot{V}_I$ to baseline levels during RA or HH. Naloxone methiodide did not increase V_T or breathing frequency relative to buprenorphine during HH. Data are presented as mean ± SD. Statistical analyses were performed using two-way repeated-measures ANOVA with Tukey's HSD post-hoc tests and statistical significance defined as $p < 0.05$. Significant ANOVA results are indicated in the upper left corner of each panel: Gas mixture (*); drug (#); and gas × drug interaction (†). Post-hoc comparisons are listed in Supplemental Table 1.

Supplemental Table 1. Post-hoc comparisons of respiratory parameters for experiments where buprenorphine was followed by naloxone methiodide. *P*-values shown in bold when significant ($p < 0.05$). *RA = Room air; HH = Hypoxic-hypercapnic challenge; Ctrl = Control; Bup = Buprenorphine; NM Post = Naloxone methiodide post-treatment.*

**Gas**	**Drug Comparison**	**$\dot{V}_I$**	**V_T**	**Frequency**
		p-value	*p-value*	*p-value*
RA	Ctrl vs. Bup	**0.0101**	0.400	**0.00120**
	Bup vs. NM Post	0.0518	**0.0227**	0.359
	NM Post vs. Ctrl	0.129	0.967	**0.00193**
HH	Ctrl vs. Bup	**0.00942**	**0.0467**	**0.00173**
	Bup vs. NM Post	0.563	0.556	0.931
	NM Post vs. Ctrl	**0.0391**	0.411	**0.00247**
**Drug**	**Gas Comparison**	**$\dot{V}_I$**	**V_T**	**Frequency**
		p-value	*p-value*	*p-value*
Ctrl	RA vs. HH	**0.00508**	**0.00546**	**0.00554**
Bup	RA vs. HH	**0.0299**	**0.0438**	0.0614
NM Post	RA vs. HH	0.0593	0.0665	0.617

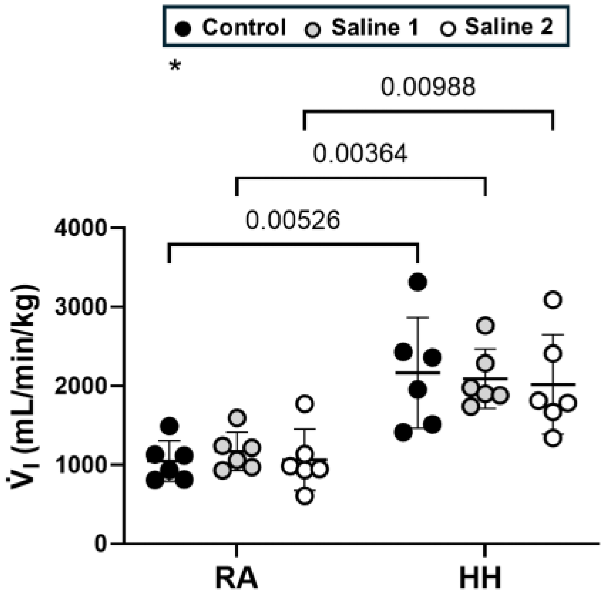

Supplemental Figure 2. Ventilation is unaffected by sham injections and remains stable across repeated trials. In time and sham-controls, $\dot{V}_I$ was measured using dual-chamber plethysmography in neonatal Sprague Dawley rats (n = 6; 3 males; P4-5) during RA breathing and a HH challenge. Measurements were obtained at baseline, and after each of two sham injections (Saline 1, Saline 2) over a time course matched to Experiments I-III. Data are shown as individual replicates with mean ± SD for each gas and treatment condition. Two-way repeated measures ANOVA revealed a significant effect of gas mixture ($F_{1,10} = 45.19$, $p = 0.00110$), but no effect of saline injection. $\dot{V}_I$ consistently increased in response to HH, with no difference between trials. Significant ANOVA results are indicated in the upper left corner: Main effect of gas mixture (*). Significant post-hoc comparisons (Tukey's HSD) are shown on the plot. The threshold for statistical significance was $p < 0.05$.

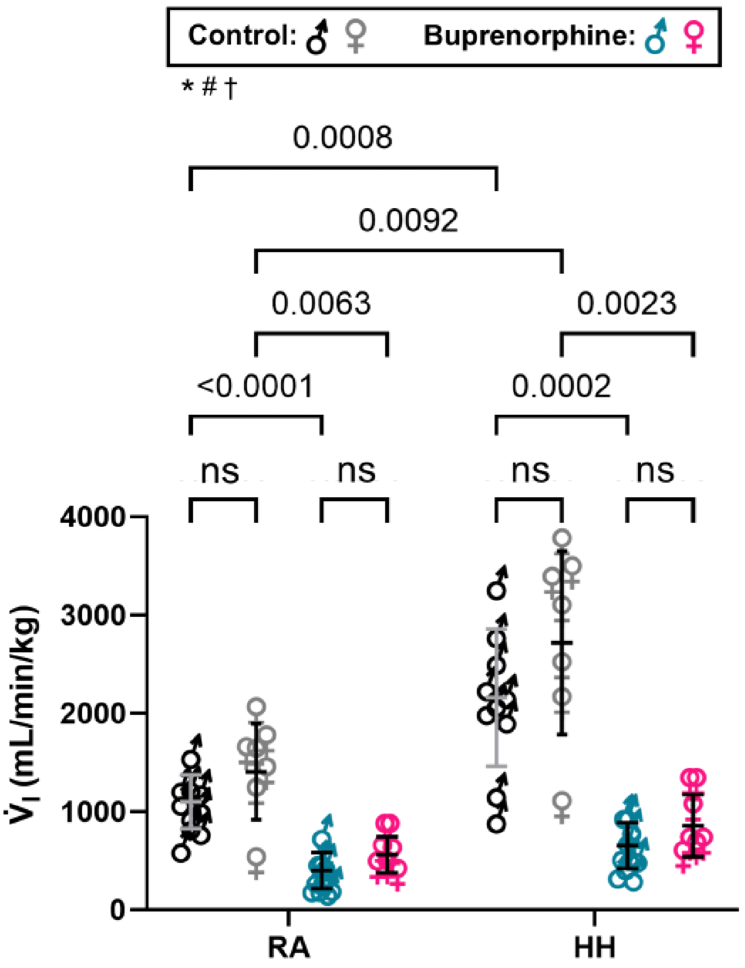

Supplemental Figure 3. Acute buprenorphine depresses the neonatal hypoxic-hypercapnic ventilatory response independent of sex. $\dot{V}_I$ was measured in male (*n* = 10) and female (*n* = 7) P4–5 Sprague Dawley rat pups during RA breathing and a HH challenge at baseline and following buprenorphine administration. Data are shown as individual values with mean ± SD for both sexes under each gas and treatment condition. A three-way repeated-measures ANOVA revealed significant effects of gas mixture ($F_{1,15} = 88.89$, $p < 0.0001$), drug ($F_{1,15} = 111.4$, $p < 0.0001$), and a gas × drug interaction ($F_{1,15} = 53.89$, $p < 0.0001$). There was no significant effect of biological sex. At baseline, $\dot{V}_I$ increased significantly in response to HH in both males and females. Following buprenorphine, $\dot{V}_I$ was reduced during RA and HH in both males and females. Significant ANOVA effects are indicated in the upper left corner: Gas

mixture (*); drug (#); gas × drug interaction (†). Significant post-hoc comparisons (Tukey's HSD) are shown on the plot. The threshold for statistical significance was $p < 0.05$.
